# Incidental Tentorial Cerebelli Hypoplasia: A Case Report and Comprehensive Literature Review of Its Clinical and Imaging Characteristics

**DOI:** 10.1155/carm/2788624

**Published:** 2026-06-28

**Authors:** Husam Hamshary, Mosub Qatu, Fathi Milhem, Radi Kukhun, Zaid Sawaftah, Mohammad Bdair, Abdelfattah M. Dahmas, Qutaiba Ja’far A. Mahmoud, Omar Naseef, Mohammed Ibrahim Abu Kamesh

**Affiliations:** ^1^ Department of Medicine, An Najah National University, Nablus, State of Palestine, najah.edu; ^2^ Department of Radiology, An Najah National University Hospital, Tubas, State of Palestine

**Keywords:** brain herniation, CNS malformations, congenital anomalies, neuroimaging findings, tentorial hypoplasia

## Abstract

**Background:**

Tentorial hypoplasia is a rare congenital condition characterized by underdevelopment or absence of the tentorium cerebelli, a dural structure separating the cerebrum from the cerebellum. It is often detected incidentally on computed tomography (CT) or magnetic resonance imaging (MRI) and may be associated with conditions such as Dandy–Walker syndrome and Chiari malformation, although many cases remain asymptomatic. Imaging plays an essential role in identifying associated abnormalities when present.

**Case Presentations:**

Case 1: A 16‐year‐old male presented with a left subdural hematoma following a road traffic accident. Neuroimaging incidentally revealed tentorial hypoplasia with brain herniation into the quadrigeminal cistern, though he remained asymptomatic. Case 2: A 3‐year‐old child with metastatic neuroblastoma had incidentally discovered tentorial hypoplasia with brain herniation on CT and MRI, with no neurological deficits.

**Conclusion:**

Tentorial hypoplasia is often an incidental finding with a favorable prognosis, though early imaging is essential for identifying associated central nervous system (CNS) malformations and guiding management.

## 1. Introduction

Tentorial hypoplasia is an uncommon congenital condition that results from either incomplete or total underdevelopment of the tentorium cerebelli, a dural fold and the second largest among the four distinctive dural partitions [1]. The tentorium cerebelli has the transverse sinus in its composition and serves to divide the cranial cavity into the supratentorial and infratentorial segments [2]. Its role involves supporting the temporal and occipital lobes while reducing pressure on the cerebellum to ensure a normally structured cranial cavity [2].

Tentorial hypoplasia is commonly discovered through neuroimaging, being recognized as a highly unusual entity, especially when occurring alone [2]. While many are asymptomatic, some of the symptoms that have been recorded include headaches, vertigo, seizures, forgetfulness, and cognitive impairment [2, 3]. Various prevalence studies suggest a higher incidence rate among females [2, 4]. This disease can be associated with extreme central nervous system anomalies such as Dandy–Walker malformation, Arnold–Chiari malformation, and Gorlin syndrome or appear independently as a minor anomaly, which is extremely rare [3]. It has also been observed in both autopsy studies and medical imaging techniques such as computed tomography (CT) and magnetic resonance imaging (MRI) scans [1].

This study presents two cases of this rare incidental finding, tentorial hypoplasia, discussing its anatomy, imaging findings, clinical presentation, and management.

## 2. Case Presentation

### 2.1. Case 1

An adolescent male patient, 16 years old, was admitted after a road traffic accident in which he was hit by a car while riding a bicycle. He had multiple injuries, with the predominance of head injuries. He was hemodynamically stable on admission with the Glasgow Coma Scale score equal to 15 out of 15 points. The patient had a history of idiopathic thrombocytopenic purpura (ITP); there was no other history of either surgical interventions or relevant familial diseases.

Cranial CT imaging revealed the presence of a left parietal epidural hematoma accompanied by moderately depressed and comminuted left parietal bone fractures, with an additional subgaleal hematoma. The follow‐up imaging demonstrated the progression of the hemorrhage with an additional development of a left subdural hematoma; consequently, emergency surgery and hematoma evacuation were performed. Apart from the above mentioned findings, CT also revealed the presence of soft tissue herniation through the right tentorium cerebelli (Figure 1).

**FIGURE 1:**
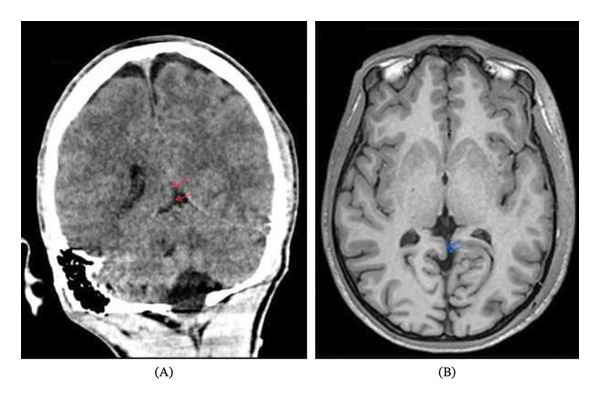
Panel (A) depicts a coronal CT scan, while panel (B) presents an axial MRI. Both images reveal right‐sided preuncal gyrus herniation through the right tentorium cerebelli into the quadrigeminal cistern, indicative of idiopathic brain herniation, suggesting congenital tentorial hypoplasia.

In order to get further confirmation of this finding, brain MRI was performed. Brain MRI revealed the presence of brain herniation into the right quadrigeminal cistern due to the hypoplastic right‐sided tentorium cerebelli (Figure 1). No neurological symptoms such as headache, vertigo, seizures, syncope, visual disturbances, tremors, or memory loss were described by the patient. The postoperative period passed without complications, with discharge of a stable patient in the absence of neurological defects. Further assessment demonstrated full recovery with no clinical features related to the tentorial anomaly.

### 2.2. Case 2

A 3‐year‐old patient was referred from another institution for further examination of the presence of paraaortic abdominal and bilateral cervical lymphadenopathy. There was no significant medical or surgical history or positive family history regarding malignancies. Histopathological examination confirmed the presence of metastases of the neuroblastoma originating from paraaortic abdominal primary neuroblastoma.

During the staging procedure, whole body CT imaging of the brain, chest, abdomen, and pelvis was performed. Brain CT revealed the presence of tentorial hypoplasia and right‐sided preuncal brain herniation in the quadrigeminal cisterns, without any presence of intracranial masses or metastatic lesions (Figure 2). Additional brain MRI confirmed the findings of CT imaging (Figure 2).

**FIGURE 2:**
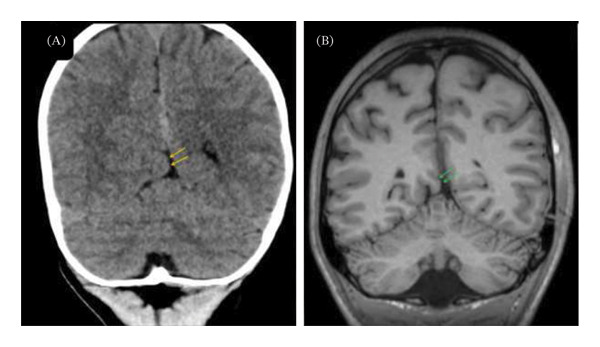
Panel (A) depicts a coronal CT scan, while panel (B) presents an axial MRI. Both images demonstrate evidence of cingulate and precuneus gyrus herniation through the right tentorium cerebelli into the quadrigeminal cistern on the right side. These findings are consistent with incidental brain herniation due to congenital tentorial hypoplasia.

No neurological symptoms or signs of increased intracranial pressure were found in the patient, which suggests that these findings are incidental ones. High‐risk neuroblastoma was confirmed, with the subsequent administration of chemotherapy using irinotecan and temozolomide; six chemotherapy cycles were administered to the patient without major side effects or neurological complications.

## 3. Discussion

The tentorium cerebelli is an arched membrane which separates the supratentorial compartment from the infratentorial compartment and provides structural support for the posterior cranial fossa. It has free and fixed edges, where the midbrain passes through the tentorial notch, located between the free edges of the membrane. Fixed edges anchor to the anterior clinoid process and the groove of the transverse sinus on the occipital bone [3]. Its physiological functions include stabilization of intracranial structures and protection against displacement of the brain stem due to fluctuating intracranial pressure [5].

Tentorial hypoplasia is classified as a very rare form of congenital disorder that is characterized by the underdevelopment or thinning of the tentorium cerebelli. It can allow herniation of nearby cerebral gyri into the quadrigeminal cistern. In most cases, this condition is diagnosed accidentally through CT and MRI conducted to examine other underlying issues [3, 6]. Tentorial hypoplasia has been shown to occur in conjunction with many congenital anomalies of the posterior cranial fossa, like the Chiari II malformation and the Dandy–Walker malformation, and some syndromic entities such as Aicardi syndrome and Miller–Dieker syndrome [3, 7, 8].

It is still not fully known what causes this congenital condition. The pathophysiological mechanism behind the development of tentorial hypoplasia can include abnormal mesoderm development in the early embryo stage, defective dural folding, vascular injuries, and brain development abnormalities of genetic origin. Disturbances in normal development in the period between sixth and tenth week of intrauterine life might lead to the incomplete formation of the tentorium cerebelli. Other proposed causes of this disease are changes in cerebrospinal fluid dynamics and apoptosis in the neural development process [3, 8].

MRI scanning is considered the imaging method of choice for detection of tentorial hypoplasia. It is more accurate than CT in terms of soft tissue contrast, which allows revealing associated gyral herniation and other intracranial disorders. Also, CT may be helpful for diagnosis of this condition, especially in emergency situations [6, 9, 10]. Recent prevalence studies using MRI suggest that tentorial hypoplasia could be underdiagnosed, with a prevalence of about 3% in routine brain MRI scans and that associated gyral herniation is often asymptomatic. Similarly, previous prevalence studies have shown that females are more commonly affected than males [4]. Interestingly, both our patients were males. Furthermore, both patients had unilateral right‐sided involvement, despite the fact that bilateral involvement has been previously reported in the literature [4, 6]. Similar to previously reported cases, neither of our patients demonstrated neurological symptoms attributable to the tentorial defect [11].

Differential diagnoses for tentorium cerebelli hypoplasia, particularly in cases involving occipital lobe herniation, include Chiari malformation, cerebral atrophy, hydrocephalus, Dandy–Walker malformation, and syndromic conditions such as Aicardi syndrome and Miller–Dieker syndrome [3, 11].

Management of tentorium cerebelli hypoplasia is primarily conservative and tailored to the severity of the condition and associated neurological deficits. Key approaches include the treatment of hydrocephalus using a ventriculoperitoneal (VP) shunt to divert cerebrospinal fluid and alleviate intracranial pressure. Physical, occupational, and speech therapy may be required for motor or speech deficits, and early intervention programs can significantly enhance developmental outcomes. Surgical interventions may be necessary for associated malformations, such as Chiari malformation or Dandy–Walker syndrome, to decompress the brainstem or correct structural abnormalities. Regular follow‐up with imaging is essential to monitor for complications and ensure the effectiveness of interventions [3]. In our case report, both patients had a stable neurological state and were asymptomatic concerning the lesion in the tentorium, which confirms the good prognosis seen in tentorial hypoplasia in isolation.

## 4. Conclusion

In conclusion, tentorial hypoplasia is a rare condition that is often diagnosed incidentally, typically through imaging studies. While it can be associated with severe CNS malformations, it can also occur as an isolated anomaly with minimal symptoms. When identified, imaging allows evaluation for associated anomalies, although isolated tentorial hypoplasia is typically benign. Although the condition may remain asymptomatic in many cases, awareness of its potential impact on brain structures and function is essential for proper clinical management and long‐term outcomes. Regular follow‐up and imaging are key for monitoring any developments.

## Funding

No funding was received for this research.

## Ethics Statement

Our institution does not require ethical approval for reporting individual cases or case series.

## Consent

Written informed consent was obtained from the patients for their anonymized information to be published in this article.

## Conflicts of Interest

The authors declare no conflicts of interest.

## Data Availability

Data sharing is not applicable to this article as no datasets were generated or analyzed during the current study.
